# Evaluation of medical student retention of clinical skills following simulation training

**DOI:** 10.1186/s12909-019-1663-2

**Published:** 2019-07-16

**Authors:** Gozie Offiah, Lenin P. Ekpotu, Siobhan Murphy, Daniel Kane, Alison Gordon, Muireann O’Sullivan, Sue Faye Sharifuddin, A. D. K. Hill, Claire M. Condron

**Affiliations:** Department of Surgery, Royal College of Surgeons in Ireland Education and Research Centre, Beaumont Hospital, Dublin 9, Ireland

**Keywords:** Simulation, Retention, Clinical skills, Medical school curriculum, Psychomotor and cognitive domains, Deliberate practice

## Abstract

**Background:**

Adequate clinical skills training is a challenge for present day medical education. Simulation Based Education (SBE) is playing an increasingly important role in healthcare education worldwide to teach invasive procedures. The impact of this teaching on students along with retention of what is taught is not fully understood. The purpose of this study was to evaluate the retention levels of practical skills taught and assessed by SBE and to explore the degree of re-training required to restore decayed performance. In exploring this aim, the study further investigates how skilled performance decays over time and which dimensions of clinical skills were more likely to decay.

**Methods:**

Study participants were 51 final year medical students. They were provided with online pre-course videos and procedural guides asynchronously with repeatedly access. 7 of the skills taught over 2 years using task trainers were selected. Following demonstration from faculty, students practiced in small groups with faculty facilitated supervision and peer support prior to formal testing. Score sheets with itemised procedure checklists detailing the minimum passing standard (MPS) for each skill were designed. To test retention of skills, 18 months later, there was an unannounced test to demonstrate proficiency in the skills. Students were asked to complete a questionnaire indicating how many times and where they had practiced or performed the skills.

**Results:**

55% of the students were deficient in 3 or more skills and 4% were not competent in 5 or more skills. A significant number of students had never practiced some skills following the initial teaching session. A relationship was noted with the number of times students self-declared that they had practiced and their performance. Decay is evident in both psychomotor and cognitive domains of the skills.

**Conclusion:**

A curriculum with deliberate practice significantly increases the competence of students in defined clinical skills. Deliberate practice of clinical skills, under supervision of an engaged instructor, is a key component of the mastery model. Experiences and assessments in the clinical setting need to be augmented with focus on direct observation and focused feedback to reinforce the skills acquired in the simulated setting.

## Background

Clinical skills are a key domain of good professional practice and recently there has been a greater recognition of the performance-based component of clinical competency [1]. Inadequate clinical skills training is a significant problem of present-day medical education [2]. Many procedures are potentially dangerous due to their invasive nature and thus difficult to teach and learn. Simulation based education (SBE) is playing an increasingly important role in healthcare education worldwide. In addition to reducing risk to patients, simulation is valued for the ability to create conditions that optimise learning. Intricate elements of a difficult procedure can be selectively rehearsed again and again, and learners reach competence through deliberate and repeated practice aided by timely feedback and appropriate reflection.

Engaging in deliberate practice with use of simulation, reflection and feedback develops expertise and this has been well documented in the literature [3]. Clinical competence is defined as having “the knowledge and skills for safe and effective practice when working without direct supervision” [4]. Clinical skill mastery is developmental, thus clinical skills education must be an integrated and longitudinal educational process. Several studies have shown that the use of low fidelity simulator improves both the technical performance but also the ability to attend to the cognitive components of the skill [5]. Cognitive skills training enhances the ability to correctly execute a technical skill and thus needs specific focus within the medical school curriculum [6].

## Objectives

The aim of our study was to investigate clinical skills performance over a period following SBE training in an undergraduate medical curriculum. We hypothesized that certain dimensions of clinical skills performance are more subject to decay over time. Does the cognitive learning or the technical skill learning decay more? Identifying the aspects that decay more than others will lead to designing interventions to strengthen any component skills.

## Methods

### Study design

Medical students’ skills decay was investigated in observational prospective cohort study.

### Setting

The study was carried out with final year medical students at the Royal College of Surgeons in Ireland (RCSI). Ethical approval for the study was obtained from the RCSI ethics committee (number REC 1362).

Skills training is focussed in the third, fourth and final years. A mastery learning approach was taken to teach 7 practical skills over the 2 years between third year and final fifth year to undergraduate medical students. Learning resources incorporating procedural guides and videos were provided on-line prior to the initial training and available for students to repeatedly use at any time. A faculty led programme provided skill demonstration using task trainers and facilitated mentored practice. Students practiced with faculty supervision and peer support before undergoing formal testing using a scoresheet detailing the minimum passing standard (MPS). The MPS was set by consensus from a group of educators across the different specialities and departments within the institution teaching these skills.

The initial training course provided the necessary educational experiences for repetitive practice required to reach competency and remediation when necessary.

### Participants and study size

In their final medical year, 51 students attended and consented to an unannounced test to demonstrate skills proficiency which was held between 12 and 24 months of their initial training sessions. This groups consisted of 48% females and 52% males. This was a convenience sample which was representative of the class as a whole.

### Bias

Selection bias maybe considered a limitation of this study in that students volunteered for the non-compulsory retesting. Voluntary participation was a condition of our study ethics permission and this was achieved by adding the retesting stations onto an induction session for the student’s sub-internship which is the final student clerkship in our institution. Some students were taken aback at the idea of unannounced testing and were initially reluctant to participate in retesting. However as they witnessed others classmates  participating, in the retesting and associated retraining they immediately saw the value in this opportunity and all but 2 consented to the resting of their skills.

### Data variables

Table 1 shows the list of skills that were tested and the time period between initial teaching and re-test. Although all of the students received the initial teaching and assessment of the different skills in (with in) the same term and the retesting took place within (in) one term controlling for the retention time was not possible in this convenience sample and is a limitation of this study.

**Table 1:** The skills that were tested and the time interval between teaching and re-test

Clinical Skill	Skill initially taught	Year taught	Year Re-assessed (time interval)
Venepuncture	3rd medical year	(2014/15)	2017 (24 months)
Cannulation	4th medical year	(2015/16)	2017 (12 months)
Male Catheterisation	3rd medical year	(2014/15)	2017 (24 months)
Blood Pressure	3th medical year	(2014/15)	2017 (24 months)
Sterile Field	4th medical year	(2015/16)	2017 (12 months)
Arterial Blood Gas	4th medical year	(2015/16)	2017 (12 months)
Blood Glucose	4th medical year	(2015/16)	2017 (12 months)

Students were taught clinical skills throughout their 3rd and 4th year of medical school. Students volunteered to rest their competency at these skills during their final year just before their final clerkship

The MPS tool for each skill scored performance as done well, done adequately or done poorly/not done.

Students completed a questionnaire to report when and how many times they practiced their skills following initial teaching.

### Analysis

To aid data analysis the individual elements of the minimum passing standard (MPS) for each skill were grouped into affective, psychomotor and cognitive type skills [7]. Table 2 shows the component parts of the score sheet aligned to Bloom’s Taxonomy.

**Table 2:** MPS Score Sheets aligned to Bloom’s Taxonomy

Affective/behavioural	Initial introduction Patient relationship building/Communication skills Explanation and consent
Psychomotor	Individual MPS components of task Technique specific
Cognitive	Knowledge specific Stepwise completion of task

The Individual Component tasks of the MPS score sheets were grouped and aligned to Bloom’s Taxonomy

## Results

Undergraduate medical students presented for unannounced practical skills testing using task trainer simulation immediately before their final clerkship (*n* = 51 students). 45% of student retained the MPS in all 7 skills over the 2-year period. 55% did not achieve the MPS in 3 or more skills and 4% did not achieve the MPS in 5 or more skills.

Of the 7 skills, male catheterisation was the most poorly retained skill while students performed best at venepuncture. The results demonstrate a correlation between the numbers of times that students self-declared that they had practiced the skills and a high competency rate as depicted in Table 3.

**Table 3:** Students Self-declared Intensity of Practise for Each Skill

Clinical Skill	Spearman’s rho Correlation coefficient	Never	Once	More than 5	10 or more
Aseptic Technique	0.16	24	16	3	1
Venepuncture	0.73	4	9	25	9
Blood Pressure	0.72	2	3	25	16
Arterial Blood Gas	0.83	27	18	0	2
Male Catheterisation	0.11	30	13	2	1
Cannulation	0.34	12	18	12	5
Blood Glucose Monitoring	0.09	14	15	13	5

Students filled in a questionnaire to detail how often they had practiced their skills. (*n* = 51 students). This self-report practice was correlated with the students’ performance on retest of the individual skills (Spearman’s Correlation)

**Fig. 1:**
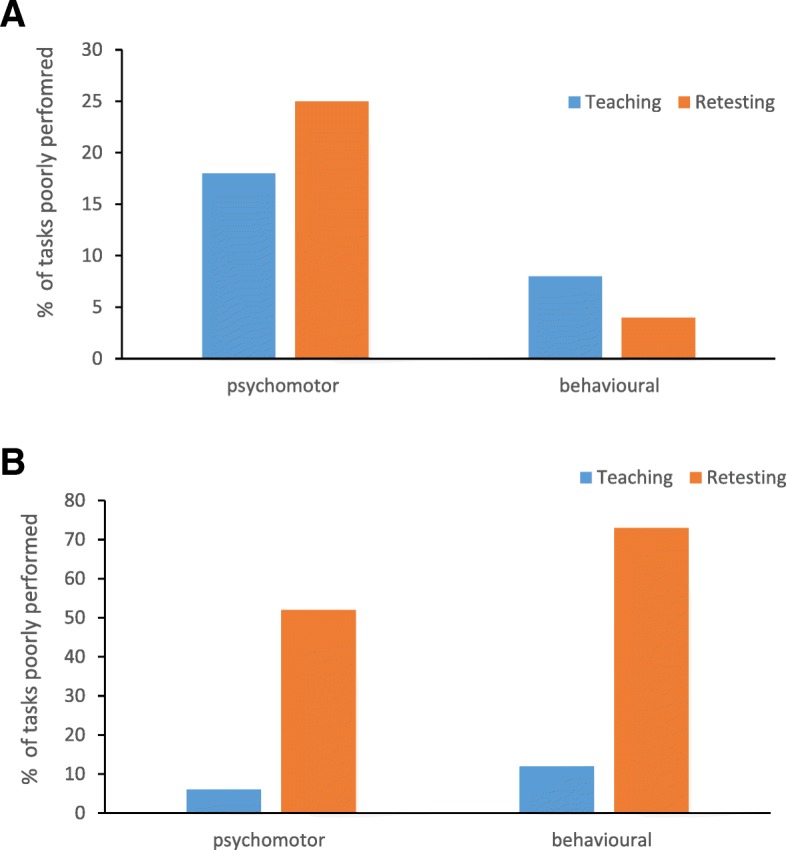
Percentage of poorly performed tasks. The individual tasks required to perform skills completely were identified under Blooms Taxonomy as psychomotor, behaviour, or knowledge. At the retest of venepuncture, students performed less poorly the behavioural aspects of this task as compared to the psychomotor elements (**a**). For catheterisation student performed equally poorly on both the psychomotor and behavioural aspects of the tasks (**b**)

Further analysis of performance of 2 of the skills with the longest interval between initial teaching and assessment and the retesting was carried out. Venepuncture which students practiced repeatedly in the intervening time and catheterisation which was practiced least by students. To facilitate this analysis the MPS were further categorised to affective, cognitive or psychomotor domains. Our result shows that different procedural skills have elements of decay from the different aspects of the blooms taxonomy domains of affective, cognitive and psychomotor when retested thus supporting the argument that deliberate repeated practice should include all components of the skills rather than just the technical aspects. It was noted that when students failed to reach the MPS at retesting the psychomotor aspects of these skills were performed poorly for both procedures. Interestingly, the behavioural aspect varied for both procedures. Students retained the behavioural aspects of the Venepuncture technique better compared to catheterisation as shown in Fig. 1.

**Fig. 2:**
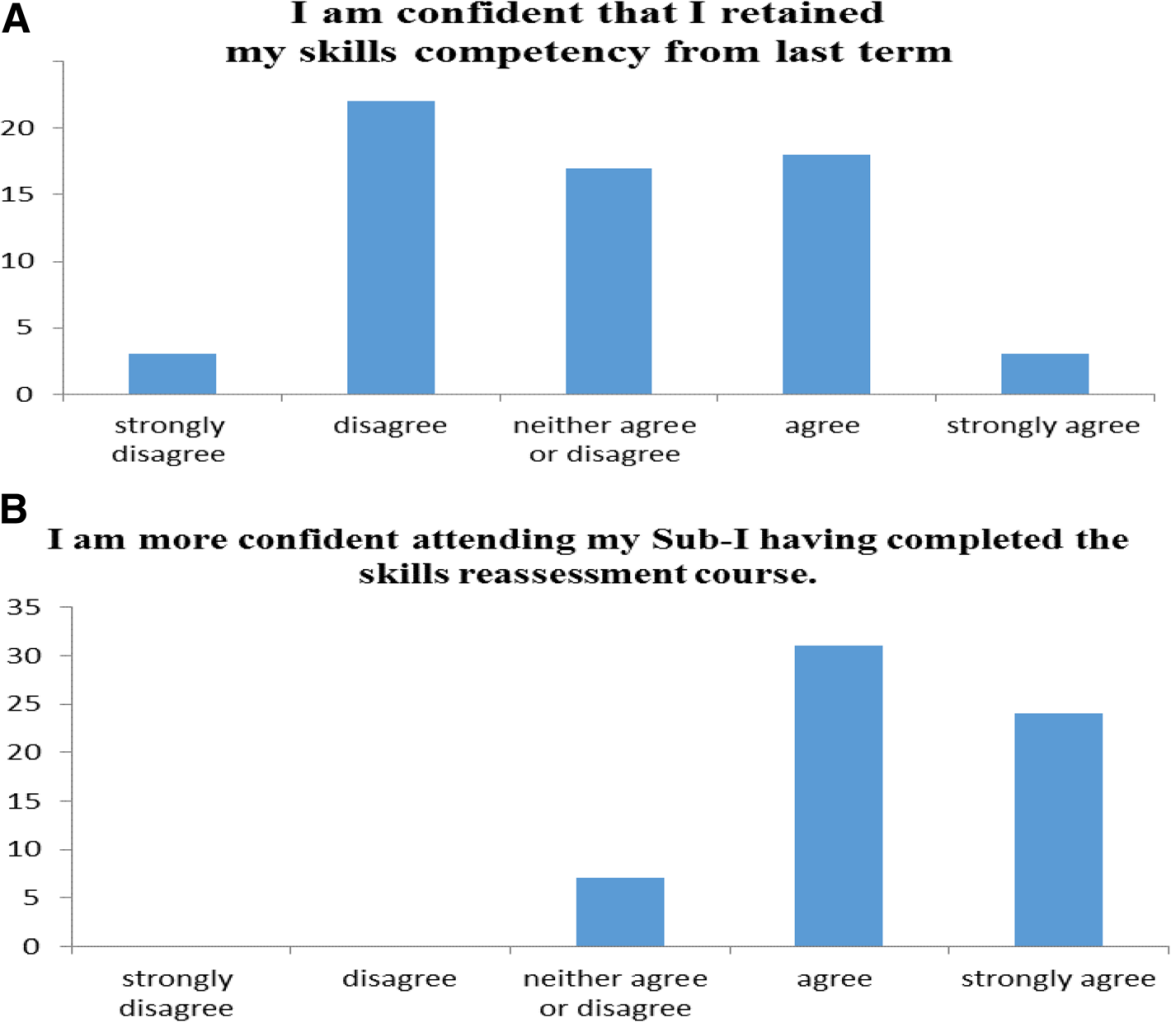
Students’ confidence in own clinical skills pre and post retesting and retraining. Students filled in a questionnaire pre and post-retesting to detail how confident they were at the individual skills. Students were not confident with their own skill levels prior to retesting (**a**) and they expressed that they were much happier to attend clinical duties post retesting (**b**). (*n* = 51 students)

Students understood the need to retest for competency and agreed that the correct time to address skills retention was before their final clerkship in our program. They felt more confident after the retest and prior to commencing their clerkship as depicted in Fig. 2. Students agreed that SBE provided a safe environment to practice skills.

Additionally, we evaluated where in the medical school curriculum these skills should be taught and the relevance for preparing medical students for clinical practice. 91 participants were asked to complete a Likert scale questionnaire. Table 4 below. Both interns (first year post graduation) and medical educators identified that these skills were required for safe intern practice yet 55% of our final year students did not reach competency at for 3 or more skills.

**Table 4:** Medical interns and Medical Educators rate essential skills required for internship

Required competency for Intern Practice	Intern	Educator	Intern	Educator	Intern	Educator	Intern	Educator	Intern	Educator
	Strongly agree		Agree		Undecided		Disagree		Strongly disagree	
Venepuncture	90%	83%	10%	15%	0%	2%	0%	0%	0%	0%
Blood Pressure	68%	95%	19%	3%	10%	2%	3%	0%	0%	0%
Arterial blood gas	97%	67%	6%	27%	0%	5%	0%	2%	0%	0%
Urinary catheterisation	94%	68%	10%	27%	0%	5%	0%	0%	0%	0%
IV Cannulation	94%	80%	10%	17%	0%	3%	0%	0%	0%	0%

Interns *n* = 31, Educators *n* = 60

Educators and interns were asked to rate their perceived level of importance of each clinical skill from a list to the clinical practice of a competent intern

## Discussion

Research shows that healthcare providers’ skill retention declines as soon as three months after training [8, 9]. Factors known to affect skills and knowledge acquisition include motivation, the learning environment [10] and the use of simulation to enhance skills retention for low-frequency procedures [11]. Simulation-based mastery learning can improve medical students’ retention of core clinical skills and deliberate practice of clinical skills under the supervision of an engaged instructor is a key component of the mastery model [12]. Low-dose high-frequency training is a competence-building approach for training which uses targeted simulation-based learning activities that are spaced over time and reinforced with structured, ongoing practice sessions promoting maximum retention of clinical skills. Our study indicates that SBE can facilitate teaching clinical skills. The key question is how low-dose high-frequency simulation can be operationalized within the constraints of large undergraduate programs to help to reduce skills decay?

It is important to define the appropriate level of performance at stages of the curriculum and establish criterion levels for different levels of proficiency from novice to mastery. The MPS for clinical skills should be designed to ensure that the affective, psychomotor and cognitive elements of each skills are captured and to ensure as much uniformity as possible in the approach to generic elements. An entrustable professional activity (EPA) is “a core unit of professional practice that can be fully entrusted to a trainee, as soon as he or she has demonstrated the necessary competence to execute the activity unsupervised” [13]. It is hypothesised the introduction of EPAs into our curriculum with emphasis on longitudinal spiral skills mastery, with increasing complexity reinforcing previous learning will motivate student to do the required repeat practice. A companion e-portfolio log book of practice, reflection and feedback should result in better retention of key skills.

Our study shows that certain dimensions of skills performance are more subject to decay with the passage of time than others. To ensure rigour and relevance an evidenced based approach to curriculum reform is essential. Simulation allows the standardised assessment of learner performance and thus facilitates specific evaluation of a curriculum by collecting evidence to determine if acceptable standards have been met [14]. Simulation can be used to reverse engineer student performance, evaluate performance gaps, and identify knowledge errors and skills deficiencies. Appropriate evaluation of simulation outcomes can close the loop for curriculum development and can facilitate continuous improvement in the educational experience and contribute to curriculum development [15]. Once identified as not competent a work through with a facilitator providing instant feedback was sufficient to restore decayed performance to proficiency on a simulated model which reflects the findings of others [16, 17].

## Conclusions and recommendations

There is significant variation among students with respect to skill decay and this relates directly to practice. Thus, continuous practice needs to be encouraged by tailored experiences and assessments in the clinical setting with emphasis on direct observation and focused feedback to reinforce the skills acquired in the simulated setting. The question remains if performance in simulation can determine undergraduate readiness for performing such procedures on actual patients? How skills practiced and assessed in simulation can be transformed safely into clinical practice needs to be defined by an EPA framework to ensure faculty make competency-based decisions on the level of supervision required by students. To ensure rigour and relevance an evidenced based approach to curriculum reform is essential. Simulation allows the standardized assessment of learner performance and thus facilitates specific evaluation of a curriculum by collecting evidence to determine if acceptable standards have been met. The student body becomes an element in the curriculum reform process as participation provides understanding of the learning that drives the performance.

## Data Availability

All data analysed during this study are included in this published article. The datasets used and/or analysed during the current study are available from the corresponding author on reasonable request.

## Abbreviations

MPS: Minimal Passing Standard
RCSI: Royal College of Surgeons in Ireland
SBE: Simulation Based Education
